# Risk of fractures in individuals with eosinophilic esophagitis: nationwide population-based cohort study

**DOI:** 10.1007/s10388-022-00929-2

**Published:** 2022-06-28

**Authors:** John J. Garber, Bjorn Roelstraete, Paul J. Lochhead, Amiko M. Uchida, Karl Michaëlsson, Ola Olén, Jonas F. Ludvigsson

**Affiliations:** 1 Gastrointestinal Unit, Massachusetts General Hospital, Harvard Medical School, Boston, MA USA; 2 Department of Medical Epidemiology and Biostatistics, Karolinska Institutet, Stockholm, Sweden; 3 Clinical and Translational Epidemiology Unit, Mongan Institute, Massachusetts General Hospital, Harvard Medical School, Boston, MA USA; 4 Division of Gastroenterology, Hepatology and Nutrition, University of Utah School of Medicine, Salt Lake City, UT USA; 5 Department of Surgical Sciences, Uppsala University, Uppsala, Sweden; 6 Clinical Epidemiology Division, Department of Medicine Solna, Karolinska Institutet, Stockholm, Sweden; 7 Department of Clinical Science and Education Södersjukhuset, Karolinska Institutet, Stockholm, Sweden; 8 Department of Paediatrics, Örebro University Hospital, Örebro, Sweden

**Keywords:** Steroids, Eosinophilic esophagitis, Fracture, Osteoporosis, Population-based

## Abstract

**Background and aims:**

Eosinophilic esophagitis (EoE) is an emerging, chronic immune-mediated disease for which swallowed topical steroids and proton pump inhibitors (PPIs) represent first-line treatments. Immune-mediated diseases, steroids, and PPI use have been linked to osteoporosis. We assessed the risk of fractures in patients with EoE and determined whether the most commonly used treatments for EoE were associated with increased fracture risk.

**Methods:**

We followed a nationwide cohort of 1263 individuals in Sweden with biopsy-verified EoE diagnosed between 2005 and 2016 for first-time fracture of any type. Age- and sex-matched reference individuals were retrieved from the Total Population Register (*n* = 5164). We estimated hazard ratios (HRs) for fracture in relation to EoE diagnosis, steroid exposure, and PPI use. In a separate analysis, we compared fracture risk among individuals with EoE to their siblings (*n* = 1394).

**Results:**

During 4521 person-years of follow-up, 69 individuals with EoE experienced a first-time fracture (15.3/1000 person-years) compared with 234 reference individuals (12.6/1000 person-years). After adjusting for age, sex, birth year, and county of residence, EoE was not associated with a statistically significantly increased risk of fractures (HR = 1.2, 95% CI = 0.9–1.6). Among EoE individuals, exposure to PPIs and swallowed steroids did not modify the risk of fracture (p for heterogeneity 0.20 and 0.07 respectively). There was no increased risk of fractures in EoE compared to EoE-free siblings.

**Conclusion:**

The risk of fracture in EoE was not statistically significantly elevated compared to non-EoE reference individuals. Fracture risk in EoE was not modified by PPIs or steroid use.

**Supplementary Information:**

The online version contains supplementary material available at 10.1007/s10388-022-00929-2.

## Introduction

Eosinophilic esophagitis (EoE) is a chronic immune-mediated disease, which causes significant impairment in quality of life due to dysphagia and food impaction that result from progressive esophageal fibrosis [1, 2]. EoE is most often diagnosed in children and adults before the age of 50 years [3], with an overall prevalence approaching 1 in 1000 [2]. Given the life-long nature of this increasingly recognized condition, a major focus has been on understanding the range of esophageal and extra-esophageal manifestations and complications [4, 5], as well as accurately describing the true burden of the disease in terms of both economic costs [6] and impairment in quality of life [6, 7].

Proton pump inhibitors (PPIs), topical (swallowed) steroids and attempts at identifying underlying dietary trigger(s) through an empiric elimination diet represent first-line treatments for EoE. PPIs and swallowed steroids are typically given as repeated courses or long-term maintenance regimens [8, 9]. PPI therapy alone can reduce symptoms and normalize histology in approximately 50% of patients with symptomatic esophageal eosinophilia [10]. Long-term use of PPIs has been associated with increased risk of fractures [11], possibly through reduction of gastric acidity and malabsorption of calcium and vitamin B12.

Among gastrointestinal diseases, both celiac disease [12] and inflammatory bowel disease (IBD) [13] are associated with an increased risk of fractures. In IBD, the fracture risk has been directly linked to cumulative steroid exposure [13]. The use of both inhaled and systemic steroids for asthma, a closely related allergic disease that shares important genetic and environmental overlaps with EoE [14], has also been associated with decreased bone mineral density (BMD) and increased risk of fractures [15–18].

Additionally, periostin, a secreted extracellular matrix protein that is involved in regulating bone formation and turnover, is also one of the most highly differentially expressed genes in EoE [19, 20] and asthma, where serum levels have been proposed as a biomarker for severe allergic disease [21]. In the bone, periostin plays a key role in regulating osteoblast function and bone formation and remodeling [22]. Although its specific role in airway and gastrointestinal allergic inflammation is not yet fully understood, periostin is increasingly recognized as a potential link between bone metabolism and the allergic immune response [23].

Although both fluticasone and budesonide, two common swallowed steroids used for EoE treatment, are generally thought to have low systemic exposure due to first-pass metabolism [24], to date, no studies have examined the overall risk of fractures in EoE, and no data are available regarding how chronic steroids, a mainstay of current EoE treatment, affect fracture risk. We therefore performed a nationwide study encompassing more than 1200 individuals with EoE, 5164 matched reference individuals randomly selected from the general population, and 1394 siblings of EoE patients to test the hypothesis that EoE is associated with increased risk of fractures and that the risk is further increased among EoE individuals exposed to steroids and/or PPIs.

## Methods

### Setting

This study used data from Sweden’s health and welfare databases. Sweden has a tax-funded healthcare system with universal access independent of socioeconomic status and income [25, 26]. In Sweden, all residents are assigned a unique personal identity number [27] that allows for large-scale linkages of healthcare registers. In this study, we retrieved medical data from Sweden’s 28 Pathology Registers, the Patient Register [28, 29], and the Prescribed Drug Register [30]. Follow-up including date of emigration and death was ascertained through the Total Population Register [31].

### EoE

From October 12, 2015 to April 10, 2017, we contacted all pathology departments in Sweden through the ESPRESSO cohort (Epidemiology Strengthened by histoPathology Reports in Sweden) [32]. We asked them to identify all individuals with EoE defined as having a biopsy report from the esophagus with a morphology code of eosinophilia (Systematized Nomenclature of Medicine (SNOMED) system (M4715). Using current consensus guidelines, which require compatible symptoms and ≥ 15 eosinophils per high-powered field (HPF), [33] this case definition had a positive predictive value of 89% when validated by medical record review [34]. While there were reports of EoE in Sweden prior to 2004, these were rare and may represent severe disease that was registered in retrospect. We therefore restricted this study to biopsy reports from 2005 to 2016.

### Education levels

Level of education was divided into three groups (compulsory school ≤ 9 years, upper secondary school 10–12 years, college or university ≥ 13 years). Data were retrieved from the LISA database (Longitudinal Integrated Database for Health Insurance and Labour Market Studies) [35]. A fourth group was created for people with missing education levels.

### Reference individuals

#### General population

Each individual with EoE was matched with up to five reference individuals from the Total Population Register according to age at time of diagnosis, sex, calendar year of diagnosis, and county of residence [36]. Reference individuals had to be free from EoE at time of matching (date of first biopsy) but could develop EoE in the future (if so, their follow-up as reference individuals was censored).

#### Siblings

Sibling analyses enable researchers to adjust for shared genetic and environmental factors and thus reduce residual confounding. EoE-free siblings of individuals with EoE (*n* = 1394) were identified through the Total Population Register and Multigeneration Register [36]. Sibling data were available on all individuals born after 1932 and who were registered as residents of Sweden after 1961.

#### Steroids and proton pump inhibitors

The Swedish Prescribed Drug Register [30] began in July 2005. From here, we obtained data on dispensed prescriptions for both systemic and locally acting steroids (the corresponding Anatomic Therapeutic Chemical (ATC) codes are listed in Supplementary Table 1). We divided steroid use into ever (1 or more prescriptions) or never exposed. Swallowed (topical) steroids are considered a first-line treatment for EoE, and we included the most commonly used steroids, fluticasone, budesonide, cicelsonide, mometasone, and beclomethasone [8, 37–39]. Additionally, we identified subjects who received the following PPIs: omeprazole, pantoprazole, lansoprazole, rabeprazole, esomeprazole, dexlansoprazole, and dexrabeprazole (Supplementary Table 1).

### Fractures

In our main analysis, we examined the rate of the first outpatient or inpatient treated fracture in individuals without a prior fracture (“fracture-free individuals”) according to the Swedish Patient Register. We defined fractures according to relevant International Classification of Diseases (ICD) codes (Supplementary Table 2), and have previously confirmed near complete capture of fractures and that the diagnosis of fracture of any type is highly accurate in the Swedish Patient Register [40, 41]. We also performed a separate sensitivity analysis which included individuals with a fracture prior to the start of follow-up.

### Statistics

Follow-up started at the first biopsy, from 2005 onward, for EoE individuals and the corresponding dates in the reference individuals (general population and siblings), and ended with first fracture, emigration, death, or end of follow-up (December 31, 2016), whichever happened first. Reference individuals were censored if they developed EoE (such individuals would first contribute person-time to the reference cohorts, and then to the EoE cohort).

We used Cox regression to estimate hazard ratios (HRs) for first-time fracture. The proportional hazards assumption was checked by visual inspection and by creating interaction terms with time. No evidence of violation was observed. These analyses were controlled for matching variables (sex, age at index date, county, year at index date) to remove any confounding effect they have on the risk of fracture. In a sensitivity analysis we excluded anyone with a diagnosis of hypereosinophilia, asthma or chronic obstructive pulmonary disease at baseline (Supplementary Table 2). We also performed a separate sensitivity analysis where we adjusted for celiac disease and IBD (definitions included in Supplementary Table 2) since these diseases may be more common in EoE and have been linked to fracture risk [12, 13].

We examined fracture risk according to follow-up time since EoE diagnosis and corresponding date of matching (< 1 year of follow-up, 1- < 5 years, ≥ 5 years). We also calculated HRs separately according to age, and calendar period of first EoE biopsy. Absolute risks per 1000 person-years and excess risks per 1000 person-years were also calculated.

Medication analyses (steroids and PPIs, see Supplementary Table 1 for relevant ATC codes), were limited to individuals with biopsies on January 1, 2006 or later (to allow for at least 6 months of prior exposure; the Prescribed Drug Register stared on July 1, 2005) and their matched reference individuals. First drug use needed to be administered ≤ 7 days before biopsy or after biopsy (but before first fracture). We first separately estimated fracture risk in EoE individuals with any steroid exposure during follow-up and EoE individuals without steroid exposure, comparing both to their respective matched reference individuals (and similarly for PPI use). To test for heterogeneity of the risks between medication exposed and unexposed EoE groups, we ran models comparing EoE individuals to reference individuals where an interaction term for EoE status and steroid use was included in the Cox model (an identical analysis was performed to evaluate for heterogeneity according to PPI use). We also compared the risk of fracture among EoE individuals who used both steroids and PPIs, as well as those who had used neither, to the general population. The p-value of the interaction term was used as an indication for risk heterogeneity.

Statistics were performed using R statistical software (version 3.3.1, R Foundation for Statistical Computing, Vienna, Austria) and the survival package (version 2.38, Therneau, T (2015), https://CRAN.R-project.org/package=survival). 95% CIs outside 1.0 were considered statistically significant.

### Ethics

The Ethics Review Board of Stockholm, Sweden, approved this study and waived personal consent since none of the study participants was contacted and the study was strictly register-based [42].

## Results

### Background data

A total of 1263 individuals with EoE and 5164 matched reference individuals were identified by linking the ESPRESSO cohort and population registries. Baseline characteristics of the cohorts are presented in Table 1, and for study participants entering the study on July 1, 2006 or later in Supplementary Table 1a. The median age at the start of follow-up for EoE individuals was 39 years (IQR 19–53; range 0–94 years) compared to 38 (IQR 19–52) for reference individuals. Half of the EoE patients were between 18 and 49 years old at the start of follow-up, with 22% being < 18 years and about 30% being ≥ 50 years old. Consistent with previous reports [2, 43], we found an approximately 3:1 male to female predominance, with 943 males with EoE (74.5%) and 320 females with EoE (25.5%) in our study.

**Table 1:** Summary statistics for EoE patients, reference individuals, and unaffacted siblings

	EoE	Reference individuals	Siblings
	*n* [%]	*n* [%]	*n* [%]
Total	1263 [100.00]	5164 [100.00]	1394 [100.00]
Male	943 [74.66]	3804 [73.66]	691 [49.57]
Female	320 [25.34]	1360 [26.34]	703 [50.43]
Age at start of follow-up			
Mean [SD] years	37.53 [20.50]	36.89 [20.44]	36.63 [19.68]
Median [IQR] years	39.00 [19.00–53.00]	38.00 [19.00–52.00]	38.00 [21.00–51.00]
< 18 years	284 [22.49]	1202 [23.28]	298 [21.38]
18 < 50 years	596 [47.19]	2458 [47.60]	700 [50.22]
> = 50 years	383 [30.32]	1504 [29.12]	396 [28.41]
Years of follow-up			
Mean [SD] years	3.58 [2.37]	3.59 [2.40]	3.65 [2.34]
Median [IQR] years	3.13 [1.79–4.96]	3.11 [1.76–5.13]	3.25 [1.87–5.27]
< 1 years	144 [11.40]	627 [12.14]	153 [10.98]
1 < 5 years	805 [63.74]	3215 [62.26]	870 [62.41]
> = 5 years	314 [24.86]	1322 [25.60]	371 [26.61]
Start of follow-up			
2005–2010	245 [19.40]	1020 [19.75]	284 [20.37]
2011–2013	458 [36.26]	1861 [36.04]	522 [37.45]
2014–2016	560 [44.34]	2283 [44.21]	588 [42.18]
Reason for end of follow-up			
Emigration	11 [0.87]	86 [1.67]	4 [0.29]
December 31, 2016	1166 [92.32]	4767 [92.31]	1310 [93.97]
Diagnosed with EoE	0 [0.00]	2 [0.04]	5 [0.36]
Death	17 [1.35]	75 [1.45]	16 [1.15]
Fracture	69 [5.46]	234 [4.53]	59 [4.23]
Country of birth			
Nordic	1195 [94.62]	4288 [83.04]	1336 [95.84]
Other	68 [5.38]	876 [16.96]	58 [4.16]
NA	0 [0.00]	0 [0.00]	0 [0.00]
Education			
Compulsory school (< = 9 years)	181 [14.33]	877 [16.98]	180 [12.91]
Upper secondary school (10–12 years)	450 [35.63]	1796 [34.78]	509 [36.51]
College or university (> = 13 years)	398 [31.51]	1386 [26.84]	435 [31.21]
NA	234 [18.53]	1105 [21.40]	270 [19.37]

*NA* not available

The median duration of follow-up for individuals with EoE was 3.1 years (IQR 1.8–5.0), with 24.9% being followed for ≥ 5 years (Table 1). Consistent with increased awareness of EoE starting in 2012, the majority (> 90%) of those with EoE diagnoses began follow-up in 2011 or later, with a smaller number starting follow-up between 2005 and 2010 (19.4%). The vast majority of EoE patients and reference individuals were still being followed at the end of the study period, on December 31, 2016 (92% of cases and 92% of reference individuals).

Level of education was similar among individuals with EoE and reference individuals. Among individuals with EoE, 14.3% had a record of compulsory school (≤ 9 years), 36% had attended upper secondary school (10–12 years) and 31.5% college or university (≥ 13 years) (Table 1).

### Fracture risk

**Fig. 1:**
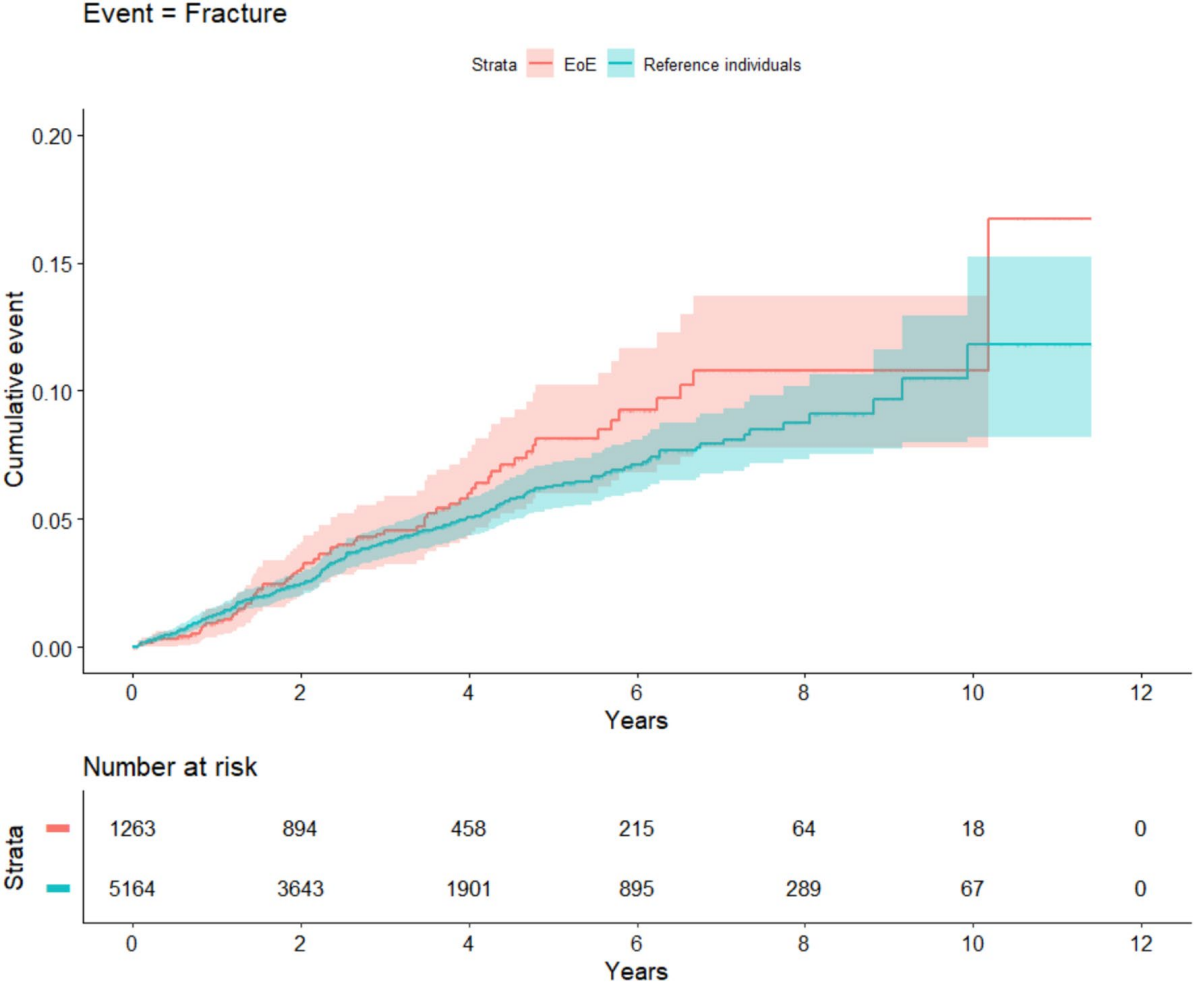
Kaplan–Meier curves of cumulative fracture events among EoE and reference individuals. Shaded areas represent 95% confidence intervals

During 4521 person-years of follow-up of individuals with EoE, there were 69 individuals with first-time fractures vs. 234 individuals over 18,522 person-years of follow-up in the matched general population reference group (Fig. 1). The median age at fracture diagnosis was 27 years for those with EoE and 34 years for reference individuals. This translated to fracture incidence rates among EoE and reference individuals of 15.3 and 12.6/1000 person-years, respectively (Table 2). EoE was not statistically significantly associated with risk of future fractures (HR = 1.2; 95% CI = 0.9–1.6) (Table 3). Excluding individuals with an earlier record of hypereosinophilia, asthma or chronic obstructive pulmonary disease yielded a similar HR (1.2; 95% CI = 0.9–1.6). Adding celiac disease and IBD to our covariates did not change the risk estimate for fracture risk (HR = 1.2; 95% CI = 0.9–1.5).

**Table 2:** Fracture incidence rates for EoE patients, reference individuals, and unaffected siblings

	EoE	Reference individuals	Siblings
*N* Total	1263	5164	1394
*N* fracture events	69	234	59
Incidence proportion (%)	5.46	4.53	4.23
Person years	4521	18,522	5084
Incidence rate/1000 person-years [95% CI]	15.26 [12.07–19.07]	12.63 [11.12–14.30]	11.61 [9.01–14.75]
Sex			
Males	14.71 [11.17–19.06]	12.62 [10.88–14.57]	15.59 [11.38–20.92]
Females	16.93 [10.88–25.34]	12.67 [9.87–16.04]	7.94 [5.21–11.67]
Age at start of follow-up			
< = 17 years	24.56 [17.04–34.46]	19.43 [15.87–23.58]	20.20 [13.61–29.04]
18 < = 49 years	10.26 [6.74–15.10]	8.89 [7.11–11.00]	7.28 [4.68–10.90]
> = 50 years	14.98 [9.74–22.23]	12.53 [9.84–15.75]	12.44 [7.70–19.23]
Years of follow-up			
< 1	9.19 [5.18–15.37]	12.73 [9.94–16.09]	15.96 [10.48–23.48]
1 < 5	18.84 [14.35–24.35]	13.37 [11.39–15.61]	11.69 [8.46–15.80]
> = 5	11.34 [5.60–21.16]	9.29 [6.26–13.36]	2.91 [0.90–8.10]
Start of follow-up			
2005–2010	15.61 [10.76–22.03]	12.81 [10.45–15.56]	12.14 [8.19–17.46]
2011–2013	15.97 [11.21–22.17]	12.60 [10.33–15.25]	10.75 [7.19–15.57]
2014–2016	13.15 [7.59–21.57]	12.35 [9.27–16.16]	12.40 [7.15–20.33]
Start of follow-up (max 2 years follow-up)			
2005–2010	18.78 [10.01–32.89]	13.53 [9.32–19.09]	16.17 [8.62–28.32]
2011–2014	13.34 [8.48–20.17]	12.10 [9.52–15.19]	12.03 [7.64–18.18]
Country of birth			
Nordic	15.83 [12.50–19.81]	13.24 [11.56–15.09]	11.70 [9.04–14.93]
Other	4.45 [1.08–16.42]	9.15 [6.22–13.07]	9.38 [2.90–26.14]
Education			
Compulsory school (< = 9 years)	20.36 [11.75–33.40]	9.38 [6.55–13.09]	9.42 [4.42–18.32]
Upper secondary school (10–12 years)	13.58 [9.00–19.81]	11.13 [8.83–13.87]	8.52 [5.27–13.18]
College or university (> = 13 years)	8.18 [4.61–13.68]	9.78 [7.34–12.80]	9.17 [5.50–14.55]
NA	24.82 [16.73–35.69]	20.24 [16.44–24.68]	22.07 [14.76–31.96]

*NA* not available

**Table 3:** Adjusted fracture hazard ratios for EoE patients, reference individuals, and unaffected siblings

	EoE	Reference individuals	Siblings
		HR [CI]	HR [CI]
Total	69/4.52	1.21 [0.92–1.58]	1.23 [0.82–1.86]
Sex			
Males	50/3.40	1.17 [0.85–1.60]	0.75 [0.42–1.34]
Females	19/1.12	1.29 [0.77–2.17]	2.24 [0.63–7.94]
Age at start of follow-up			
< = 17 years	28/1.14	1.23 [0.80–1.88]	1.24 [0.65–2.39]
18 < = 49 years	21/2.05	1.14 [0.70–1.85]	1.36 [0.60–3.08]
> = 50 years	20/1.33	1.23 [0.74–2.03]	0.98 [0.39–2.44]
Years of follow-up			
< 1	11/1.20	0.72 [0.38–1.37]	0.35 [0.13–0.97]
1 < 5	51/2.71	1.40 [1.02–1.93]	1.76 [1.07–2.91]
> = 5	7/0.62	1.53 [0.64–3.66]	0.40 [0.01–13.35]
Start of follow-up			
2005–2010	27/1.73	1.21 [0.79–1.86]	0.78 [0.36–1.67]
2011–2013	30/1.88	1.27 [0.84–1.92]	1.42 [0.76–2.67]
2014–2016	12/0.91	1.05 [0.56–1.98]	1.26 [0.51–3.14]
Start of follow-up (max 2 years follow-up)			
2005–2010	9/0.48	1.39 [ 0.65–2.96]	0.88 [ 0.31–2.52]
2011–2014	18/1.35	1.10 [0.65–1.85]	0.99 [0.46–2.14]
Country of birth			
Nordic	68/4.30	1.22 [0.92–1.60]	1.18 [0.77–1.81]
Other	1/0.22	0.56 [0.05–6.26]	
Education			
Compulsory school (< = 9 years)	12/0.59	1.66 [0.69–4.01]	2.39 [0.36–15.95]
Upper secondary school (10–12 years)	22/1.62	1.65 [0.94–2.89]	1.47 [0.57–3.79]
College or university (> = 13 years)	11/1.34	0.98 [0.44–2.18]	0.59 [0.20–1.77]
NA	24/0.97	1.19 [0.76–1.87]	1.12 [0.58–2.18]

Adjusted for sex, age, county and year of birth

*NA* not available

When patients with fractures prior to start of follow-up were included, we identified 99 EoE patients (17.8/1000 person-years) and 430 reference individuals (15.8/1000 person-years) with any fracture occurring during follow-up. This corresponded to an HR for EoE patients versus reference individuals of 1.1 [95% CI = 0.9–1.4].

Restricting our data to individuals without a prior fracture, the incidence of first-time fracture was similar in males (14.7/1000 person-years) and females (16.9/1000 person-years) and similar also to that observed among reference individuals (12.6/1000 person-years for both males and females). The highest incidence of fractures among EoE patients was in those diagnosed at age < 18 years or younger (24.6/1000 person-years), and in those with a follow-up between 1 and 5 years. These incidence rates however were not significantly different from those of the reference individuals.

Fracture risk among individuals with EoE did not differ according to education level. Several reports have described associations between low socioeconomic status and vitamin D insufficiency, lower bone mineral density and higher risk of fragility fractures [44]. In our study, we used education as a proxy for socioeconomic status and found that the risk of fractures among individuals with EoE was similar across educational strata. For individuals with EoE for whom the highest level of education was ≤ 9 years, risk of fracture was 1.6 [95% CI 0.6–4.0] compared to reference individuals (Table 3).

### Medications and fracture risk

#### PPI

Among the 1257 individuals diagnosed with EoE since January 2006, 397 (32%) had a first prescription for PPI during follow-up. Compared to the matched reference population adjusting for sex, age, calendar year and county of residence, the HR for fractures in EoE individuals exposed to PPI was 0.9 (95% CI = 0.5–1.6; based on 17 fractures in 397 EoE patients exposed to PPI). The corresponding HR for those with EoE who never received PPI compared to their matched reference individuals was 1.4 (95% CI = 1.0–1.9; based on 52 fractures in 860 EoE patients). The estimates for fracture risk associated with EoE diagnosis did not statistically significantly differ according to PPI exposure status (p for heterogeneity = 0.20) (Table 4).

**Table 4:** Summary of fracture incidence by treatment

Fractures	*N* fractures/reference individuals	*N* fractures/EoE cases	HR [95% CI]	*P* heterogeneity
No steroids	155/3430	47/844	1.23 [0.89–1.71]	
Steroids (any)	75/1708	22/413	1.21 [0.75–1.95]	0.96
Topical	51/1330	17/323	1.40 [0.81–2.43]	
Systemic	40/679	8/165	0.80 [0.37–1.71]	0.24
Both	16/301	3/75	0.77 [0.22–2.65]	
No PPI	155/3494	52/860	1.39 [1.01–1.90]	
PPI	75/1644	17/397	0.92 [0.54–1.55]	0.19
Neither	114/2374	37/588	1.32 [0.91–1.91]	
PPI and Steroids	34/588	7/141	0.82 [0.36–1.85]	0.30

#### Steroids

Among individuals with EoE, 413 were prescribed steroids during follow-up; 165 received systemic therapies (dexamethasone, methylprednisone, prednisolone, prednisone, hydrocortisone, and controlled-release budesonide), 323 received swallowed steroids (topical/inhaled), and 75 had a record of prescriptions for both topical and systemic steroids (Table 4). The most common swallowed steroids among individuals with EoE were budesonide (57.0%), fluticasone (37.0%), ciclesonide (2.2%), beclometasone (2.0%), and mometasone (1.6%). As expected, there were relatively fewer prescriptions for topical/inhaled steroids among reference individuals (1330 individuals had a prescription). Compared to reference individuals, the risk of fracture among those with EoE who received swallowed steroids was not statistically significantly elevated, (HR 1.4 [95%CI 0.8–2.4]). The same was true for EoE individuals who never received steroids (HR 1.2 [95% CI 0.9–1.7]). Again, there did not appear to be a difference in the association between EoE and fracture risk in patients with or without steroids (*p* for heterogeneity = 0.07). When analyzed separately, we did not observe a significant increase in fracture risk in individuals who were exposed to topical steroids vs. systemic steroids (HR for topical steroids alone 1.4 [95% CI = 0.8–2.4], vs. systemic steroids 0.8 [95% CI = 0.4–1.7] (p for heterogeneity = 0.2). Finally, the HR for any fracture in individuals with records of both steroids and PPIs was 0.8 (95% CI = 0.4–1.9), while the HR in individuals who never received steroids or PPIs was 1.3 (95% CI = 0.9–1.9) (*p* for heterogeneity = 0.3) (Table 4).

### Sibling comparisons

We identified 1394 siblings of individuals with EoE by linking to the Multigeneration Register within the Total Population Register. As expected, for siblings unaffected by EoE, the sex distribution was essentially equal (49.6% male, 50.4% female) and the median age of siblings, 38 years (IQR 21–51), was very similar to individuals with EoE (Table 1). The median follow-up for the sibling cohort was 3.3 years (IQR 1.9–5.3).

There were 59 individuals who experienced a first-time fracture among EoE siblings, corresponding to an incidence rate of 11.6/1000 person-years of follow-up, compared to 15.3/1000 person-years among individuals with EoE (Table 2). The median age of fractures among unaffected EoE siblings was 31 years compared to 27 years among individuals with EoE. The HR for fracture among individuals with EoE compared to siblings without EoE was 1.2 [95% CI 0.8–1.9] (Table 3). We did not observe any association between fracture risk and EoE, compared to non-EoE siblings when adjusting for age, calendar period of follow-up, and level of education (Table 3). Five individuals (0.4%) in the sibling reference group were eventually diagnosed with EoE. When including those with prior fractures, the HR for EoE patients versus unaffected siblings was 1.3 [0.8–2.0] and when celiac disease and IBD were included in the statistical model, the HR for EoE compared to unaffected siblings was 1.3 [0.8–2.0].

## Discussion

In this population-based study of more than 1200 individuals with EoE, we did not observe a statistically significant increased risk of fractures when compared to 5200 population reference individuals without EoE, who were matched on multiple factors including age, sex, and calendar period at start of follow-up. Among individuals with EoE, we did not find evidence to suggest that risk of fracture differs according to PPIs or steroids exposure. The risk of fracture associated with EoE diagnosis was not elevated in secondary analyses where siblings served as the referent group. To our knowledge, this is the first large-scale study exploring fracture risk in EoE.

### Main findings and comparison with earlier literature

Several chronic gastrointestinal conditions have been directly or indirectly associated with impaired bone mineral density and increased fracture risk. In a large, population-based study, celiac disease was associated with a 1.4-fold increase in fracture risk [12]. Fracture risk has also been reported to be elevated (HR = 1.18; 95% CI = 1.15–1.20) in patients with IBD [13]. Although EoE has been associated with both celiac disease [45] and IBD [46, 47], and often involves dietary restrictions (including dairy avoidance) [48], we did not find any increased risk of fractures among individuals with EoE.

### EoE medications and fractures

Steroids and PPIs, which have both been associated with decreased bone mineral density and increased fracture risk in observational studies [13, 15–17], represent first-line therapies for EoE and are often used repeatedly or as long-term maintenance therapy. The most commonly used steroids for treatment of EoE are generally formulated for asthma and chronic obstructive pulmonary disease treatment via inhalation [49, 50], and, in the context of lung disease, their use has been associated with impaired BMD [51–53]. There are important differences in the deposition, absorption and metabolism of steroids when they are inhaled (for lung disease) vs. swallowed (for EoE), and, in general, the estimated oral bioavailability of steroids that are most commonly used for EoE is thought to be low due to inefficient absorption and extensive first-pass metabolism (budesonide 11%, fluticasone < 1%, ciclesonide < 1%, mometasone < 1%) [54]. However, several studies have also demonstrated that the use of both budesonide as well as fluticasone for treatment of EoE is associated with significant rates of adrenal suppression determined by adrenocorticotropic hormone stimulation testing [55, 56]. Prior to this study, no data existed regarding the potential association between swallowed steroids and outcomes related to bone health in patients with EoE.

As expected, a significant proportion of subjects with EoE were exposed to swallowed steroids. Approximately 25% of individuals with EoE received at least one prescription for one of the commonly used topical steroids. In that subset of patients, the HR for fracture was 1.4 (albeit not statistically significant), which is numerically higher than what we found in our recent high-powered study on IBD, and similar to that for hip fracture in the same study (1.42; 95% CI = 1.36–1.48 [13]). The upper 95%CI in this subset of patients was 2.4 so a substantial increased risk of fracture could exist in the subgroup of EoE individuals exposed to steroid, but we did not have statistical power to detect it. Overall, our finding that the association between EoE and fracture was not modified by steroid exposure may contrast with earlier studies, such as the meta-analysis by Kanis et al. [57] where they examined any prior exposure to corticosteroids, primarily systemic. This may be primarily due to the younger average age of individuals with EoE in our cohort, as well as the fundamental differences in absorption and metabolism of swallowed/topical steroids versus systemic therapies such as prednisolone or its equivalents.

Although PPI medications represent a highly effective therapy for a significant subset of EoE patients, their use has been associated with increased risk of hip fractures. A recent meta-analysis including 24 observational studies covering more than 2 million participants found that patients exposed to PPIs had a greater risk of hip fracture than those without PPI therapy, and the association between PPI use and increased risk of hip fracture was observed in both low and medium doses, but was greatest among patients using higher doses [58]. EoE consensus guidelines recommend a therapeutic trial of PPI for 6–12 weeks [59, 60], followed by symptom and histology reassessment. Although individualizing treatment to find the lowest minimum PPI dose capable of maintaining disease control has been examined [61], many patients with PPI-responsive EoE are maintained on long-term therapy [62, 63], and no guidelines exist regarding the overall duration of therapy or specific timeframe and criteria for tapering EoE patients off PPIs [64]. Despite these areas of uncertainty, no studies have examined the risk of fractures in EoE patients treated with PPIs.

Our study identified 397 individuals with EoE who were treated with a PPI. We did not find a difference in risk of fracture associated with EoE according to PPI use. These data are reassuring in that the benefit of PPI medications, which represent a powerful tool for reducing eosinophilic inflammation and mitigating the long-term structuring complications of EoE for nearly half of patients, appears to outweigh the potential effects on fracture risk.

Finally, the risk of fracture was not significantly increased either among EoE individuals who received both steroids and PPIs or individuals who had received neither.

### Strengths and limitations

One of the main strengths of this study is the large number of EoE patients. We found no statistically significantly increased fracture risk (HR = 1.2; 95% CI = 0.9–1.6). The tight 95%CI in our main analysis also means that we can rule out a more than 60% increased fracture risk. This should be comforting to patients with this chronic disorder. Furthermore, our large sample enabled us to perform analyses stratified by sex and age.

Data on medication use were retrieved through the Swedish Prescribed Drug Register. This register is virtually complete, with < 0.3% of records lacking the personal identity number needed for linkage with other national registers in Sweden. Through linking to the Total Population Register we were able to obtain emigration and mortality data that allowed accurate calculation of follow-up times without loss of follow-up.

EoE was identified through a computerized search for the relevant SnoMed code in 28 Swedish pathology departments. The nationwide character of the ESPRESSO study [32] means that we are likely to have avoided selection bias since both urban and rural areas were included. Thus, EoE cases should be representative of those occurring in other Western populations. Access to Swedish healthcare is tax-funded and we do not expect any bias due to socioeconomic factors. Besides, HRs did not change when we adjusted for level of education. We have previously demonstrated that our case definition has a high validity for an EoE diagnosis [34] as does ascertainment of the outcome using international classification codes for fracture. Finally, we used two different reference groups to disentangle factors of possible importance to fracture risk. In a secondary analysis we compared individuals with EoE to siblings without EoE. The use of sibling comparators helps to minimize confounding by unmeasured genetic, lifestyle, and environmental factors.

The major limitation of our study is the lack of data regarding smoking, which has been linked to osteoporosis and fractures [65], exercise level and body-mass index (BMI). We also noted a relatively low median age of EoE individuals at the start of follow-up (39 years) which is well before the main increase in fracture incidence with advancing age, and also accounts for the relatively low number of fracture events. Another major limitation of our study is that individuals were analyzed according to categories of exposed vs. never exposed to PPIs and steroids, and not based on the duration of treatment or cumulative exposure. Given the frequent need for repeated treatment courses or long-term maintenance therapy, it is likely that some therapy was administered long-term and the overall lack of increased fracture frequency is reassuring in this regard. We also did not have data on disease activity in EoE, and we cannot rule out that more severe EoE may be associated with risk of fractures, nor did we have the statistical power to examine subgroups of fractures. Finally, due to lack of data, we were unable to examine the impact of elimination diet on fracture risk in EoE and data on the frequency of esophageal dilation therapy were not available.

### Clinical implications

Currently, no guidelines exist regarding osteoporosis screening in patients with EoE, or among patients treated with PPIs or swallowed steroids. EoE patients who are prescribed swallowed steroids or PPIs may raise concerns about whether these potentially affect their bone health. Our data suggest that the risk of fractures among individuals with EoE is not substantially elevated compared to the general population, and medications commonly used to treat EoE do not appear to modify that risk among those with EoE. Our findings will be reassuring for EoE patients who ask about the risk of fractures when treated with swallowed steroids or PPI.

## Conclusion

The risk of fracture in EoE was not significantly elevated compared to non-EoE reference individuals. We did not find statistical evidence that PPI or steroid use modified the risk of fracture associated with EoE. Nevertheless, we cannot exclude the possibility that use of steroids among individuals with EoE is associated with a moderately higher risk of fracture.

## Supplementary Information

Below is the link to the electronic supplementary material.

Supplementary file1 (DOCX 13 KB)

Supplementary file2 (DOCX 14 KB)

Supplementary file3 (DOCX 27 KB)
