# Supplementary material for: Risk of fractures in individuals with eosinophilic esophagitis: nationwide population-based cohort study
Source: Esophagus. 2022 Jun 28;19(4):542–53. doi: 10.1007/s10388-022-00929-2 (PMC9436880; doi:10.1007/s10388-022-00929-2)
Supplement: Supplementary file 1 — Supplementary file1 (DOCX 13 KB) [file 10388_2022_929_MOESM1_ESM.docx]

**Supplementary Table 1**. ATC codes for corticosteroids and PPI

**Corticosteroid ATC code**

*Systemic*

betamethasone H02AB01

dexamethasone H02AB02

methylprednisolone H02AB04

prednisolone H02AB06

prednisone H02AB07

hydrocortisone H02AB09

cortisone H02AB10

*Swallowed/topical*

budesonide R03BA02

ciclesonide R03BA08

fluticasone R03BA05

mometasone R01AD09

beclomethasone R03BA01

**PPI ATC code**

omeprazole A02BC01

pantoprazole A02BC02

lansoprazole A02BC03

rabeprazole A02BC04

esomeprazole A02BC05

dexlansoprazole A02BC06

dexrabeprazole A02BC07
