# Supplementary material for: Risk of fractures in individuals with eosinophilic esophagitis: nationwide population-based cohort study
Source: Esophagus. 2022 Jun 28;19(4):542–53. doi: 10.1007/s10388-022-00929-2 (PMC9436880; doi:10.1007/s10388-022-00929-2)
Supplement: Supplementary file 2 — Supplementary file2 (DOCX 14 KB) [file 10388_2022_929_MOESM2_ESM.docx]

**Supplementary Table 2**. International classification disease (ICD) codes for fracture.

|  | *ICD 7, 8, 9* | *ICD 10* |
| --- | --- | --- |
| *Fracture* | *800 - 829* | *S02, S12, S22, S32, S42, S52, S62, S72, S82, S92, T02, T12* |

|  | *ICD 7* | *ICD 8* | *ICD 9* | *ICD 10* |
| --- | --- | --- | --- | --- |
| *IBD* | *572,20 572,21 578,03 572,00 572,09* | *563,10 563,99 569,02 563,00* | *556 555* | *K50 K51 K523* |
| *Hypereosinophilia* | *-* | *-* | *-* | *D47.4* |
| *COPD including asthma* |  | *490-496* | *490-496* | *J40-J47* |

|  | *TOPO* | *SNOMED* |
| --- | --- | --- |
| *Celiac disease* | *T64 T65* | *M58 M5800 M58000 M58001 M58005 M58006 M58007 M58000 XD6218 D62180 D6218X D6218Y D62188'* |

*ICD 7-9*

*800-804 Fracture of Skull*

*805-809 Fracture of Spine and Trunk*

*810-819 Fracture of Upper Limb*

*820-829 Fracture of Lower Limb*

*ICD10*

*S02 Fracture of skull and facial bones*

*S12 Fracture of cervical vertebra and other parts of neck*

*S22 Fracture of rib(s), sternum and thoracic spine*

*S32 Fracture of lumbar spine and pelvis*

*S42 Fracture of shoulder and upper arm*

*S52 Fracture of forearm*

*S62 Fracture at wrist and hand level*

*S72 Fracture of femur*

*S82 Fracture of lower leg, including ankle*

*S92 Fracture of foot and toe, except ankle*

*T02 Fractures involving multiple body regions*

*T12 Fracture of lower limb, level unspecified*
